# Ongoing Mycophenolate Treatment Impairs Anti-SARS-CoV-2 Vaccination Response in Patients Affected by Chronic Inflammatory Autoimmune Diseases or Liver Transplantation Recipients: Results of the RIVALSA Prospective Cohort

**DOI:** 10.3390/v14081766

**Published:** 2022-08-12

**Authors:** Erika Zecca, Manuela Rizzi, Stelvio Tonello, Erica Matino, Martina Costanzo, Eleonora Rizzi, Giuseppe Francesco Casciaro, Giulia Francesca Manfredi, Antonio Acquaviva, Ileana Gagliardi, Elisa Calzaducca, Venkata Ramana Mallela, Davide D’Onghia, Rosalba Minisini, Mattia Bellan, Luigi Mario Castello, Francesco Gavelli, Gian Carlo Avanzi, Filippo Patrucco, Annalisa Chiocchetti, Mario Pirisi, Cristina Rigamonti, Daniele Lilleri, Daniele Sola, Pier Paolo Sainaghi

**Affiliations:** 1 Department of Translational Medicine, Università del Piemonte Orientale (UPO), 28100 Novara, Italy; 2 COVID-19 Unit, Department of Internal Medicine, AOU “Maggiore della Carità”, 28100 Novara, Italy; 3 COVID-19 Sub-Intensive Unit, Division of Emergency Medicine, AOU “Maggiore della Carità”, 28100 Novara, Italy; 4 Internal Medicine and Rheumatology Unit, AOU “Maggiore della Carità”, 28100 Novara, Italy; 5 Center for Autoimmune and Allergic Diseases (CAAD), Università del Piemonte Orientale (UPO), 28100 Novara, Italy; 6 Division of Internal Medicine, Azienda Ospedaliera “SS. Antonio e Biagio e Cesare Arrigo”, 15100 Alessandria, Italy; 7 Medical Department, Division of Respiratory Diseases, AOU “Maggiore della Carità”, 28100 Novara, Italy; 8 Department of Health Sciences, Università del Piemonte Orientale (UPO), 28100 Novara, Italy; 9 Unit of Microbiology and Virology, Fondazione IRCCS Policlinico San Matteo, 27100 Pavia, Italy

**Keywords:** anti-SARS-CoV-2 vaccination, immunosuppressive therapy, mycophenolate, calcineurin inhibitors, autoimmune diseases, liver transplant

## Abstract

Vaccines are the most effective means to prevent the potentially deadly effects of SARS-CoV-2 infection, but not all vaccinated individuals gain the same degree of protection. Patients undergoing chronic immunosuppressive therapy due to autoimmune diseases or liver transplants, for example, may show impaired anti-SARS-CoV-2 antibody response after vaccination. We performed a prospective observational study with parallel arms, aiming to (a) evaluate seroconversion after anti-SARS-CoV-2 mRNA vaccine administration in different subgroups of patients receiving immunosuppressive treatment for rheumatological or autoimmune diseases or to prevent organ rejection after liver transplantation and (b) identify negative predictors of IgG anti-SARS-CoV-2 development. Out of 437 eligible patients, 183 individuals were enrolled at the Rheumatology and Hepatology Tertiary Units of “Maggiore della Carità” University Hospital in Novara: of those, 52 were healthy subjects, while among the remaining 131 patients, 30 had a diagnosis of spondyloarthritis, 25 had autoimmune hepatitis, 10 were liver transplantation recipients, 23 suffered from connective tissue diseases (including 10 cases that overlapped with other diseases), 40 were treated for rheumatoid arthritis, and 5 had vasculitis. Moreover, all patients were receiving chronic immunosuppressive therapy. The immunogenicity of mRNA COVID-19 vaccines was evaluated by measuring IgG anti-SARS-CoV-2 antibody titers before vaccination and after 10, 30, and 90 days since the first dose administration. Of the selected cohort of patients, 24.0% did not develop any detectable anti-SARS-CoV-2 IgG after a complete mRNA-based two doses primary vaccination cycle. At univariate analysis, independent predictors of an absent antibody response to vaccine were a history of liver transplantation (OR 11.5, 95% CI 2.5–53.7, *p* = 0.0018), the presence of a comorbid active neoplasia (OR 26.4, 95% CI 2.8–252.4, *p* = 0.0045), and an ongoing immunosuppressive treatment with mycophenolate (MMF) (OR 14.0, 95% CI 3.6–54.9, *p* = 0.0002) or with calcineurin inhibitors (OR 17.5, 95% CI 3.1–99.0, *p* = 0.0012). At multivariate analysis, only treatment with MMF (OR 24.8, 95% CI 5.9–103.2, *p* < 0.0001) and active neoplasia (OR 33.2, 95% CI 5.4–204.1, *p* = 0.0002) were independent predictors of seroconversion failure. These findings suggest that MMF dose reduction or suspension may be required to optimize vaccine response in these patients.

## 1. Introduction

Severe acute respiratory syndrome coronavirus 2 (SARS-CoV-2) is a coronavirus responsible for coronavirus disease 2019 (COVID-19), a very heterogeneous disease ranging from a nearly asymptomatic state to a life-threatening condition with high in-hospital mortality, with the highest toll among the elderly or those with chronic diseases and cancer [1,2,3,4]. COVID-19, in severe cases, may evolve into respiratory and multiorgan failure due to uncontrolled cytokine production, leading to massive endothelial activation and a prothrombotic condition [5,6,7]. In spite of a huge research effort, until now, a definitive explanation of these unfavorable events is lacking, and pharmacological therapies to treat severe COVID-19 have partial efficacy, so the most effective intervention to fight this pandemic is vaccination.

Since 2020, more than 50 vaccines against COVID-19 have been developed and tested in phase II and III trials [8,9,10,11]. At the beginning of the mass vaccination campaign in Europe, and particularly in Italy, the European Medicines Agency (EMA) and the Agenzia Italiana del Farmaco (AIFA—an Italian drug agency) approved anti-COVID-19 vaccines that were based on either recombinant messenger ribonucleic acid (mRNA), such as BNT162b2 (Pfizer-BioNTech, Mainz, Germany) and mRNA-1273 (Moderna, Cambridge, MA, USA), or partially inactivated adenovirus vectors such as ChAdOx1-S (Astra-Zeneca, University of Oxford, UK) and Ad.26.COV2.S (Janssen/Johnson and Johnson, New Brunswick, NJ, USA) [8,12]. To date, the two approved mRNA-based vaccines (BNT162b2 and mRNA-1273) are intended for intramuscular administration, and different research groups reported that mRNA vaccines (those mostly used) are able to induce strong B- and T-cell immune responses, which could assure long-lasting protection against the target epitope [13,14] and are able to induce IgA and neutralizing antibody production also in the nasal district, thus supporting the role of mRNA-based vaccines in inducing mucosal immunity and finally in contributing to reduce asymptomatic transmission risk [15,16].

Patients affected by chronic immune-mediated inflammatory diseases have high rates of morbidity and mortality due to infections that rely on immunodeficiency, related to either the autoimmune diseases themselves or to the immunosuppressive drugs used to treat these illnesses [17]. Thus, the 2019 European Alliance of Associations for Rheumatology (EULAR) guidelines highlighted the importance of influenza and pneumococcal vaccination for the majority of patients affected by autoimmune inflammatory rheumatic diseases, due to the higher risk of severe infections in this population compared with the risk posed to the general population [18]. Thus far, a large number of studies and reviews have provided evidence of the safety of these vaccines [19], although transient disease flares may occur, and a lower immune response can be expected compared with the general population [19,20,21]. The availability of different vaccine formulations allowed the beginning of a massive vaccination campaign. As in many other countries, the vaccination campaign in Italy initially prioritized subjects at higher risk for adverse outcomes of SARS-CoV-2 infection, including those with autoimmune and autoinflammatory disorders, immunodeficiencies, and tumors, as well as transplanted patients [8,22,23]. Transplanted patients were also included in the patients prioritized for COVID-19 vaccination, as they undergo immunosuppressive therapies to avoid organ rejection [24].

To date, few data are available on the efficacy of anti-COVID-19 vaccination in patients with inflammatory autoimmune diseases or in transplanted patients receiving immunosuppressive treatments. To fill this gap, we performed a prospective observational study to assess seroconversion rates after vaccine administration in different subgroups of patients undergoing chronic immunosuppressive treatment due to autoimmune diseases or liver transplantation.

## 2. Patients and Methods

### 2.1. Patients

We performed a prospective observational study (acronym RIVALSA) with parallel arms including healthy subjects and autoimmune/liver-transplanted patients receiving immunosuppressive drugs with planned COVID-19 vaccination between March and July 2021. Inclusion and exclusion criteria are reported in Table 1.

**Figure 1:**
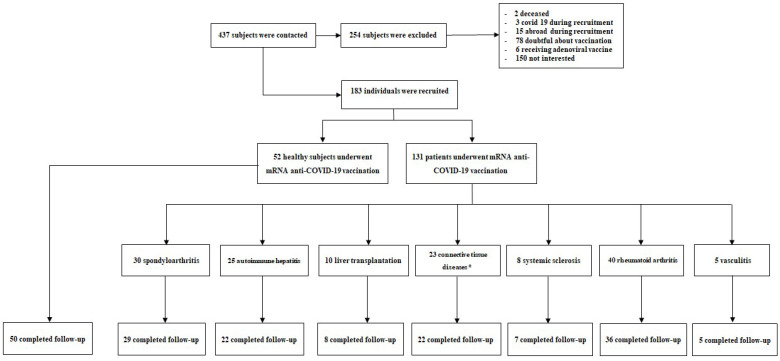
Study design. Flowchart of patient enrollment and study conduction. * Ten patients have overlapping diseases.

The study protocol was approved by the local ethical committee (CE 72/21), and the study was conducted in strict accordance with the Declaration of Helsinki. Among all the patients attending either the Rheumatology or Hepatology Unit of “Maggiore della Carità” University Hospital in Novara (Italy), eligible subjects (patients receiving immunosuppressive drugs to treat autoimmune diseases or to prevent organ rejection after liver transplantation) were asked to participate in the study; from the 437 subjects screened, 131 patients were included in the study together with 52 healthy subjects selected among the healthcare professionals of these units and/or their relatives. Figure 1 reports the details of the enrollment procedures and study conduction. Patients underwent BNT162b2 (Pfizer-BioNTech) or mRNA-1273 (Moderna) vaccination in clinical practice, according to the local protocol and vaccination schedule (primary vaccination cycle was considered completed after the second dose of each vaccine administered 21 days apart for BNT162b2 vaccine and 28 days apart for the mRNA-1273 vaccine).

### 2.2. Endpoint Definition

The predefined endpoints of the cohort study were as follows:

(1) Assessment of the IgG anti-SARS-CoV-2 titer in response to vaccination in patients who completed the primary vaccination cycle receiving immunosuppressive therapy due to inflammatory autoimmune diseases or organ rejection prevention after liver transplantation and proportion of the failure of IgG seroconversion relative to healthy subjects in patients who completed the primary vaccination cycle, 90 days after the first vaccine dose;
(2) Identification of predictors of the absence of an IgG anti-SARS-CoV-2 seroconversion 90 days after the first vaccine dose in patients who completed the primary vaccination cycle.

### 2.3. Blood Sample Collection

All individuals enrolled in the study underwent blood sampling before anti-SARS-CoV-2 vaccination (t0) and after 10, 30, and 90 days from the first vaccination dose (t10, t30, and t90, respectively). Blood samples were collected using EDTA as an anticoagulant, and blood fractions were immediately separated via centrifugation and stored at −80 °C until the time of analysis.

### 2.4. Anti-SARS-CoV-2 Antibody Quantification

IgG anti-spike protein antibodies were determined by performing an ELISA test, using a CE-IVD commercial kit (EUROIMMUN Medizinische Labordiagnostika AG Anti-SARS-CoV-2 QuantiVac ELISA (IgG), Lübeck, Germany) [25,26], following the manufacturer’s instructions. Briefly, this kit is able to detect both binding and neutralizing antibodies and is used for ELISA tests in which the reagent wells are coated with the recombinant S1 domain of the spike protein of SARS-CoV-2. These tests have been shown to correlate well with the conventional as well as surrogate neutralizing antibody assays [27,28,29]. Prior to quantification, plasma samples were diluted at 1:101 ratios in a dilution buffer (provided by the manufacturer). The absorbance value was recorded using a Victor X4 microplate reader (PerkinElmer, Waltham, MA, USA). Optical density at 450 nm was fitted versus a point-to-point calibration curve prepared using human IgG (0–120 RU/mL).

### 2.5. Data Collection and Statistical Analysis

The relevant clinical and experimental data of each subject (demographics, therapy, comorbidities, previous SARS-CoV-2 infection, and IgG anti-spike protein quantification) were collected from clinical records or during an ad hoc interview and stored in a dedicated database (REDCap). Categorical variables were expressed as frequencies (percentage), while the measures of central tendency and dispersion of continuous variables were, respectively, expressed as medians and interquartile range (IQR). Categorical variables were compared with Pearson’s χ^2^ test or Fisher’s exact test when appropriate, while continuous variables were compared with the Mann-Whitney U test. Odds ratios (ORs) with confidence intervals (CIs) were calculated at univariate analysis. A set of variables associated with seroconversion failure at univariate analysis were used to build multivariate stepwise logistic regression models. The statistical significance threshold was set at 0.05 (two-tailed). Statistical analyses were performed with Statistica for Windows release 12 (TIBCO Software Inc., Palo Alto, CA, USA) and MedCalc^®^ Statistical Software version 20.014 (MedCalc Software Ltd., Ostend, Belgium).

## 3. Results

Among the 131 patients, 69.5% were women with a median age of 58 years (IQR 49–67). Healthy controls had a median age of 32 years (IQR 29–45) and were mainly women (65.4%). Most of them were healthcare professionals and had no comorbidities.

The baseline population features (patients and controls) are shown in Table 2.

Comparing the two groups, we observed a significant difference in the median antibody titers between healthy subjects and patients at 10, 30, and 90 days since the first vaccine dose (Table 3).

This difference was also confirmed after the exclusion of those subjects with baseline antibody positivity for any time with the exception of the assessment at 90 days after vaccination (Table 4).

Interestingly, 50 out of the 50 healthy individuals with complete follow-up (100%) developed anti-SARS-CoV-2 IgG antibodies; by contrast, only 100 out of 119 patients with complete follow-up (84.0%) showed a detectable antibody response to vaccination at 90 days (Fisher’s exact test, *p* = 0.0010).

We then performed a univariate statistical analysis (Table 5) to verify which variables were associated with an absent IgG anti-SARS-CoV-2 seroconversion after vaccination. To avoid the bias of previous exposure to SARS-CoV-2, all subjects showing baseline antibody positivity were excluded from univariate and subsequent multivariate statistical analysis. A history of liver transplantation, the presence of comorbid active neoplasia, and ongoing immunosuppressive treatment with mycophenolate (MMF) or with calcineurin inhibitors were all independent predictors of vaccination failure (the detailed immunosuppressive regimen for the selected patients is shown in Supplementary Table S1).

We then built a few multivariate stepwise logistic regression models to confirm independent predictors of vaccination failure. As shown in Table 6, in one model, only variables related to comorbidities or clinical conditions were included, and based on the results, undergoing liver transplantation, having systemic lupus erythematosus (SLE), or having active cancer were all independent predictors of vaccination failure.

In a different model, in which only individual immunosuppressive drugs associated were included as independent variables, MMF and calcineurin inhibitors were the only independent predictors of vaccination failure (Table 7).

Finally, a multivariate stepwise logistic regression analysis model including all the variables associated with seroconversion failure in the two previous models (*p* < 0.05) was built. Treatment with MMF and active cancer as comorbidity were independent predictors of the absence of vaccine response in this model (Table 8).

## 4. Discussion

In this observational, parallel-group, prospective cohort study, we found that up to 24.0% of the patients affected by inflammatory autoimmune diseases or liver transplantation in treatment with immunosuppressive drugs did not have a detectable titer of IgG anti-spike protein after 10, 30, and 90 days since the first dose of anti-SARS-CoV-2 mRNA vaccine. In addition, patients had also a slight but evident longer latency in increasing IgG anti-SARS-CoV-2, which recovered at 90 days. Ongoing MMF or calcineurin inhibitors treatment, being affected by SLE or liver transplantation, and the compresence of active neoplasia as comorbidity were all independent predictors of an absent seroconversion after vaccination in different multivariate models.

It should be noted that 50% of the control population versus 13.7% of the patients displayed a detectable anti-spike IgG titer before vaccination (Table 2). These data support an active viral circulation in our district and in particular among healthcare professionals and their families, along with a probable, strong adherence of immunocompromised patients to personal protection practices.

Most current vaccines for SARS-CoV-2 show very high levels of protection, with particularly marked efficacy in relation to severe disease and death. They induce a protective clinical effect within 11 days after the first vaccination, by inducing a T-cell response devoted to both activating cytotoxic T cells and supporting the generation and maintenance of high-affinity antibodies [30].

While reduced seroconversion after vaccination in patients affected by autoimmune diseases has already been widely reported for other vaccines, scarce data are available for COVID-19 vaccination.

Our results are in line with those reported by Furer et al., in a multicenter trial that assessed the immunogenicity and safety of a two-dose regimen of BNT162b2 mRNA vaccine in 686 adults with chronic inflammatory diseases, by evaluating the IgG anti-SARS-CoV-2 titer after 2 and 6 weeks from vaccination. In this study, a negative response to vaccination was observed in 14% of patients [31]. Our study confirms a similar proportion of inadequate immunogenicity 90 days after the first dose of a complete immunization cycle.

Of great interest is the finding that, in our study, most of the immunosuppressive therapies did not influence antibody response to vaccination. In particular, neither the treatment with c-DMARDs (methotrexate, leflunomide, azathioprine, sulfasalazine, and hydroxychloroquine) nor with b-DMARDs (anti-IL6, anti-TNF, anti-IL-17, and abatacept) was associated with the absence of IgG anti-spike antibody detection. These results are even more interesting when compared with the existing literature about DMARDs and immune response to vaccines. Indeed, previous studies assessing antirheumatic drug effects on vaccine immunogenicity were mainly focused on seasonal influenza and pneumococcal vaccines. These showed reduced humoral responses in methotrexate-, abatacept-, and rituximab-treated patients and a minimal or absent response in patients treated with anti-cytokine drugs. Such data thus raise the question about the effectiveness of anti-SARS-CoV-2 vaccination in DMARD-treated patients and foster new studies aimed to evaluate immunomodulatory drug effect on COVID-19 vaccination in rheumatologic patients [32,33,34,35]. To date, the results reported in the literature on this topic are limited, based on small patient cohorts, and characterized by variable diagnoses and therapeutic regimens, thus hampering definitive conclusions.

With the aim to evaluate the immunogenicity of a single dose BNT162b2 mRNA vaccine, Bugatti et al., studied 140 chronic inflammatory arthritis patients and observed that anti-cytokine drugs had a low impact on vaccination (>80% response), while the treatment with methotrexate and/or glucocorticoids had a greater effect in reducing the IgG seroconversion, even after methotrexate withholding before and after vaccination [36]. In a larger study focused on two geographically independent cohorts of immune-mediated inflammatory diseases, Haberman et al., confirmed that methotrexate had a negative impact on both cellular and humoral immune responses after the complete two-dose BNT162b2 mRNA vaccination schedule, observing a reduced production of anti-SARS-CoV-2 spike protein specific IgG, as well as a limited increase in CD8^+^ T cells in a third of patients treated with methotrexate [37]. On the other hand, Boyarsky et al., reported that methotrexate did not negatively impact antibody response [38], a result in line with our report.

Interestingly, studying a cohort of 404 rheumatic patients, Ruddy et al., showed that anti-TNF-α therapy did not influence antibody response (100%), while only 82% of patients receiving glucocorticoids and 73% receiving MMF had IgG seroconversion [39]. In our population, a low dose regimen of glucocorticoids (6.5 mg) did not influence the response to vaccination. Unfortunately, a dose-dependent effect analysis on antibody elevation was precluded given the low mean dose of glucocorticoids.

Notably, MMF and calcineurin inhibitors impaired immunogenicity to vaccination in our population as well. Indeed, the present study provided evidence of a perdurable absence of antibody response up to 3 months after the first dose administration and the following second one, suggesting a definitive absence of seroconversion. Such interesting results are in agreement not only with those of Furer et al., on autoimmune inflammatory rheumatic patients [31] but also with the observations of different independent research groups focused on transplant recipients. Such patients have a high risk of developing severe COVID-19 illness due to their need for combined immunosuppressive therapy, generally based on calcineurin inhibitors, MMF, and corticosteroids, to prevent graft rejection. Recently, in a cohort of patients undergoing cardiothoracic organ transplants, Schramm et al., observed the lack of response to the complete two-dose mRNA vaccination in patients treated with a combination of calcineurin inhibitor and MMF [40]. Similar results were observed also by other researchers [41,42] in kidney transplant recipients, whose seroconversion after two doses of mRNA vaccines was significantly lower in patients treated with such immunosuppressors. Consistently, limited humoral response to mRNA-based SARS-CoV-2 vaccine was also observed in an Israeli cohort of liver transplanted individuals treated with a double (60%) or triple (21%) immunosuppressive therapy [43].

In univariate analysis, being affected by SLE, undergoing liver transplantation, and being treated with MMF or calcineurin inhibitors were all predictors of the lack of response to vaccination. When all of these predictors were analyzed together in a comprehensive multivariate model, only MMF treatment remained associated with an increased risk of vaccination failure. Such observation is of great interest, as MMF is a common treatment used in either SLE or liver transplantation and may account for the principal lack of immunogenicity observed in these patients. The observed impaired humoral response to mRNA-based vaccines in patients treated with MMF and calcineurin inhibitors could be explained by their pharmacological mechanism of action. Both classes of drugs, in fact, are known to impair T-cell proliferation and activation processes, especially by suppressing follicular T helper cells, thus reducing B-cell-mediated antibody production [44,45,46,47,48]. A study on cell-mediated response complementing our findings is mandatory to better characterize the MMF action on individual cell types to elucidate mechanistic differences between responders and nonresponders.

As an additional point of interest, we observed that the concomitant diagnosis of neoplasia was a negative predictor of antibody response in all the analyses. This result is not surprising if we consider that, due to their therapeutic regimen, these patients show an increased risk to develop severe COVID-19 manifestations. To date, oncological patients have been generally excluded from anti-SARS-CoV-2 efficacy clinical trials, and studies assessing vaccine immunogenicity in these individuals are still few. The emerging literature on this topic highlights that seroconversion in oncological patients is significantly reduced compared with a healthy population [49,50,51].

Recently, Mitchell et al. [52] observed that immunosuppressed patients receiving mRNA-1273 vaccine developed a higher seroresponse than those receiving BNT162b2, while this difference was not observed in immunocompetent subjects. We could not evaluate this issue in our population due to the unbalanced distribution of mRNA vaccines among patients (92.4% received BNT162b2 and 7.6% mRNA-1273).

Due to the ongoing pandemic situation, and according to recent lines of evidence highlighting that vaccine efficacy decreases over time [53,54], many countries started to strongly recommend booster vaccinations, especially in frail populations, such as the elderly and severely immunosuppressed patients [53,55]. The available literature data about third-dose immunogenicity in immunosuppressed individuals show that the booster dose increases antibody production in such patients [56,57,58], even if some differences are still present according to the specific clinical condition, thus supporting the need for further studies to allow a tailored vaccination schedule for frail populations.

As the pandemic situation evolved, different SARS-CoV-2 variants emerged, acquiring new mutations that could account for immune escape properties. Such lines of evidence foster studies aimed to investigate the ability of vaccine-elicited immunity to neutralize these new threats. According to the “Istituto Superiore di Sanità” (the Italian health agency), at the time of the present study, the prevalent variant in Italy was the lineage B.1.1.7 (Alpha variant). Different research groups investigated the ability of post-immunization sera to neutralize circulating variants, highlighting that the Alpha variant is unlikely to escape neutralization through both BNT162b2- and mRNA-1273-elicited antibodies and to increase reinfection risk [59,60,61,62].

The present study has some limitations. First of all, the sample size was relatively small, and the population of patients was heterogeneous in terms of diseases, limiting the possibility to identify predictors of vaccination failure with smaller effects than those evidenced due to eventual biases. Additionally, control cohort heterogeneity could, to some extent, limit the possibility to elaborate conclusions stronger than those proposed. Furthermore, we focused only on people receiving mRNA-based vaccines, as only few of our patients received adenoviral vaccines, thus precluding the evaluation of this kind of vaccine immunogenicity. In addition, the chosen assay detected only IgG and not IgM or IgA against SARS-CoV-2 spike protein. Therefore, we cannot exclude that other antibodies targeted to other viral antigens may be present. Moreover, we did not evaluate T-cell-mediated response; thus, a deeper investigation of the cell-mediated response to viral infection would be necessary to better elucidate the role of MMF in vaccination failure. Due to the local COVID-19 infection recording protocol, not establishing a routine identification of viral variants, it was not possible to draw any conclusion about the possible role of the timely prevalent variants of concern in determining vaccination failure. Furthermore, the adopted study setting and the healthcare data availability did not allow a deeper investigation of vaccine efficacy in protecting against symptomatic infection. Finally, the monocentric design of the study may have led to some bias due to the clinical practice of our center.

## 5. Conclusions

In this prospective, observational study, we showed that a limited but relevant proportion of patients receiving immunosuppressive therapy for inflammatory autoimmune diseases or prevention of solid organ rejection after liver transplantation did not develop detectable anti-SARS-CoV-2 IgG three months after a complete, two-dose mRNA vaccine schedule. The principal predictors of the lack of seroconversion were ongoing treatment with MMF and the compresence of active neoplasia as comorbidity. Individuals under treatment with calcineurin inhibitors or those affected by SLE or undergoing liver transplantation also had a high risk of a failure of vaccine response, but the risk may be biased by concomitant MMF treatment. Our study suggests that MMF treatment modification, its temporary suspension, or a different vaccine regimen may be required in these populations to enhance immune responses.

## Figures and Tables

**Table 1:** Enrollment criteria: inclusion and exclusion criteria for patients and healthy subjects.

Patients		Healthy Subjects	
Inclusion Criteria	Exclusion Criteria	Inclusion Criteria	Exclusion Criteria
Age > 18 years old; Signed informed consent; Diagnosis of spondyloarthritis, autoimmune hepatitis, rheumatoid arthritis, connective tissue disease, vasculitis, liver transplantation; Chronic immunosuppressive therapy; Planned mRNA-based anti-SARS-CoV-2 primary vaccination cycle (2 doses of BNT162b2 or mRNA-1273 vaccines administered in clinical practice according to local healthcare policy)	SARS-CoV-2 infection during enrollment; Concomitant immunodeficiency; Unwillingness to undergo COVID-19 vaccination; Planned adenoviral-based anti-SARS-CoV-2 primary vaccination cycle (2 doses of ChAdOx1-S or a single dose of Ad.26.COV2.S vaccines administered in clinical practice according to local healthcare policy)	Age > 18 years old; Signed informed consent; Absence of a diagnosis of autoimmune disease or liver transplantation; Not receiving immunosuppressive therapy; Planned mRNA-based anti-SARS-CoV-2 primary vaccination cycle (2 doses of BNT162b2 or mRNA-1273 vaccines administered in clinical practice according to local healthcare policy)	SARS-CoV-2 infection during enrollment; Concomitant immunodeficiency; Unwillingness to undergo COVID-19 vaccination; Planned adenoviral-based anti-SARS-CoV-2 primary vaccination cycle (2 doses of ChAdOx1-S or a single dose of Ad.26.COV2.S vaccines administered in clinical practice according to local healthcare policy)

**Table 2:** Population characteristics. Baseline features of the enrolled subjects. Continuous variables are expressed as median [interquartile range]. Categorical variables are expressed as frequency (percentage).

Variable	Healthy Population (*n* = 52)	Patients (*n* = 131)
**Sex**		
*Male*	18 (34.6%)	40 (30.5%)
*Female*	34 (65.4%)	91 (69.5%)
**Median Age [IQR]**	32 [29–45]	58 [49–67]
**Comorbidities**		
*Coronary artery disease*	0 (0%)	7 (5.3%)
*Hypertension*	0 (0%)	42 (32.1%)
*Neoplasia*	0 (0%)	6 (4.6%)
*Chronic obstructive pulmonary disease*	0 (0%)	4 (3.1%)
*Pulmonary arterial hypertension*	0 (0%)	3 (2.3%)
*Thyroiditis*	0 (0%)	19 (14.5%)
*Diabetes mellitus type II*	0 (0%)	18 (13.7%)
**Vaccine**		
*BNT162b2 (Pfizer-BioNTech)*	50 (96.2%)	121 (92.4%)
*mRNA-1273 (Moderna)*	2 (3.8%)	10 (7.6%)
**Baseline positivity of IgG anti-spike protein (≥8 RU/mL)**	26 (50%)	18 (13.7%)

**Table 3:** IgG anti-SARS-CoV-2 titers in the whole population. Comparison of median IgG anti-SARS-CoV-2 titer in healthy subjects and patients at baseline and at 10, 30, and 90 days since vaccination. Antibody titer is expressed in RU/mL. Bold text highlights the statistically significant results.

Variable	Healthy Subjects (*n* = 52) (RU/mL)	Patients (*n* = 131) (RU/mL)	Z	*p*-Value
**Ab t0**	9.9 [0.0–30.2]	0.0 [0.0–0.0]	−4.9397	**0.0001**
**Ab t10**	84.4 [19.4–159.8]	0.0 [0.0–12.8]	−5.3868	**0.0001**
**Ab t30**	147.9 [132.7–162.6]	94.0 [0.0–164.0]	−3.5643	**0.0004**
**Ab t90**	149.0 [121.7–181.3]	123.7 [64.0–162.1]	−2.4091	**0.0200**

**Table 4:** IgG anti-SARS-CoV-2 titers in subjects without baseline antibody positivity. Median IgG anti-SARS-CoV-2 titer in control group and patients at t10, t30, and t90, after the exclusion of subjects with antibody positivity at baseline. Antibody titer is expressed in RU/mL. Bold text highlights the statistically significant results.

Variable	Healthy Subjects (*n* = 26) (RU/mL)	Patients (*n* = 113) (RU/mL)	Z	*p*-Value
**Ab t10**	17.5 [0.0–76.4]	0.0 [0.0–0.0]	−3.8514	**0.0001**
**Ab t30**	136.0 [84.4–145.3]	65.6 [0.0–147.6]	−2.3671	**0.0179**
Ab t90	137.2 [94.3–155.5]	112.2 [57.1–156.0]	−1.0195	0.3080

**Table 5:** Univariate analysis of vaccination failure predictors. Univariate analysis of predictors of the absence of detectable IgG anti-SARS-CoV-2 90 days after the first vaccine dose. Bold text highlights the statistically significant results. Abbreviations: AIH = autoimmune hepatitis, SSc = systemic sclerosis, RA = rheumatoid arthritis, SLE = systemic lupus erythematosus, OLT = orthotopic liver transplantation, CAD = coronary artery disease, DMII = type II diabetes mellitus, COPD = chronic obstructive pulmonary disease, PAH = pulmonary artery hypertension, PDN = prednisone, MTX = methotrexate, AZA = azathioprine, HCQ = hydroxychloroquine, MMF = mycophenolate mofetil, SSZ = sulfasalazine.

Predictors	Positive Vaccine Response	Absent Vaccine Response	Odds Ratio	95% CI	*p*-Value
AIH	18/82	4/15	1.2	0.4–4.1	0.7536
SSc	7/93	0/19	0.3	0.0–5.8	0.4414
Vasculitis	5/95	0/19	0.4	0.0–8.4	0.5890
RA	32/68	4/15	0.6	0.2–1.8	0.3456
Spondyloarthritis	27/73	2/17	0.3	0.1–1.5	0.1423
SLE	7/93	4/15	3.5	0.9–13.6	0.0651
**OLT**	3/97	5/14	11.5	2.5–53.7	**0.0018**
CAD	9/94	1/18	0.6	0.1–4.9	0.6159
Hypertension	31/69	8/11	1.6	0.6–4.4	0.3473
DM II	12/88	5/14	2.6	0.8–8.6	0.1116
**Neoplasia**	1/99	4/15	26.4	2.8–252.4	**0.0045**
COPD	2/98	1/18	2.7	0.2–31.7	0.4235
PAH	2/98	1/18	2.7	0.2–31.7	0.4235
Thyroiditis	14/86	4/15	1.6	0.5–5.7	0.4350
PDN	45/55	10/9	1.4	0.5–3.6	0.5417
MTX	29/71	2/17	0.3	0.1–1.3	0.1102
AZA	33/67	2/17	0.2	0.1–1.1	0.0654
HCQ	28/72	3/16	0.5	0.1–1.8	0.2744
**MMF**	4/96	7/12	14.0	3.6–54.9	**0.0002**
Leflunomide	6/94	0/19	0.4	0.0–6.9	0.5074
SSZ	6/94	0/19	0.4	0.0–6.9	0.5074
Abatacept	2/98	0/19	1.0	0.0–21.9	0.9948
Anti-TNF	6/94	2/17	1.8	0.3–9.9	0.4760
Anti-IL6	7/93	2/17	1.6	0.3–8.2	0.5967
Anti-IL17	5/95	1/18	1.1	0.1–9.6	0.9617
**Calcineurin inhibitors**	2/98	5/14	17.5	3.1–99.0	**0.0012**
Belimumab	2/98	0/19	1.0	0.0–21.9	0.9948

**Table 6:** Multivariate analysis of clinical predictors of vaccination failure. Multivariate stepwise logistic regression considering clinical variables. The variables entered in the model are reported in the table. SSc, AIH, vasculitis, CAD, hypertension, DM II, COPD, PAH, inflammatory bowel disease (IBD), RA, and spondyloarthritis were not included in the model. Bold text highlights the statistically significant results.

Variable	Coefficient	Standard Error	*p*-Value	Odds Ratio	95% CI
**OLT**	2.5161	0.9049	**0.0054**	12.4	2.1–72.9
**SLE**	2.0737	0.7026	**0.0032**	8.0	2.0–31.5
**Neoplasia**	2.9555	0.9808	**0.0026**	19.2	2.8–131.4

**Table 7:** Multivariate analysis of pharmacological predictors of vaccination failure. Multivariate stepwise logistic regression analysis considering immunosuppressive treatments. The variables entered in the model are reported in the table. PDN, MTX, AZA, HCQ, leflunomide, SSZ, anti-TNF, anti-IL6, anti-IL17, belimumab, and abatacept were not included in the model. Bold text highlights the statistically significant results.

Variable	Coefficient	Standard Error	*p*-Value	Odds Ratio	95% CI
**MMF**	2.6966	0.7453	**0.0003**	14.8	3.4–63.9
**Calcineurin inhibitors**	2.7782	0.9772	**0.0045**	16.1	2.4–109.2

**Table 8:** Multivariate analysis of vaccination failure predictors. Multivariate stepwise logistic regression analysis including all the predictors that reached statistical significance in the multivariate models reported in Table 6 and Table 7. The variables entered in the model are reported in the table. SLE, OLT, and calcineurin inhibitors were not included in the model. Bold text highlights the statistically significant results.

Variable	Coefficient	Standard Error	*p*-Value	Odds Ratio	95% CI
**Neoplasia**	3.5033	0.9261	**0.0002**	33.2	5.4–204.1
**MMF**	3.2091	0.7281	**<0.0001**	24.8	5.9–103.2

## Data Availability

Data are available upon reasonable request from the corresponding author.

## Supplementary Materials

The following supporting information can be downloaded at: https://www.mdpi.com/article/10.3390/v14081766/s1, Table S1: Immunosuppressive therapy.
