# Supplementary material for: Ongoing Mycophenolate Treatment Impairs Anti-SARS-CoV-2 Vaccination Response in Patients Affected by Chronic Inflammatory Autoimmune Diseases or Liver Transplantation Recipients: Results of the RIVALSA Prospective Cohort
Source: Viruses. 2022 Aug 12;14(8):1766. doi: 10.3390/v14081766 (PMC9413351; doi:10.3390/v14081766)
Supplement: Supplementary file 1 [file viruses-14-01766-s001.zip › viruses-1779747-supplementary.pdf]

viruses

Table S1. Immunosuppressive therapy. Description of the immunosuppressive regimen of the selected patients.

Drug	*N. of patients*	%
Prednisone (<10 mg/day)	55	46.2
Methotrexate	31	26.1
Azathioprine	35	29.4
Hydroxychloroquine	31	26.1
Mychophenolate	11	9.2
Leflunomide	6	5.0
Sulfasalazine	6	5.0
Abatacept	2	1.7
Anti-TNF	8	6.7
Anti-IL6r	9	7.6
Anti-IL17	6	5.0
Calcineurin inhibitors	7	5.9
Belimumab	2	1.7
